# Ribulose-1,5-bisphosphate carboxylase/oxygenase activase isoforms from diverse species show differences in oligomeric structure, thermal stability, and activity

**DOI:** 10.1007/s00249-025-01794-4

**Published:** 2025-09-11

**Authors:** Jeremy R. Keown, Serena A. J. Watkin, F. Grant Pearce

**Affiliations:** 1 Biomolecular Interactions Centre and School of Biological Sciences, University of Canterbury, Private Bag 4800, Christchurch, 8020 New Zealand; 2 School of Life Sciences, University of Warwick, Coventry, United Kingdom

**Keywords:** Rubisco activase, Rubisco, AAA+ proteins, Thermal stability, Protein structure, Enzyme

## Abstract

Rubisco activase (Rca) is critical for maintaining Rubisco activity during photosynthesis by removing inhibitory sugar phosphates through ATP hydrolysis. Despite its importance, the structural and functional diversity of Rca across species remain poorly understood. This study compares the oligomeric assembly, thermal stability, and functional activities of α- and β-isoforms of Rca from cotton, creosote, Antarctic hairgrass, and Sitka spruce, representing diverse thermal and ecological adaptations. We found that cotton and creosote Rca isoforms form highly polydisperse complexes in solution, with no evidence of discrete hexamer formation, even in the presence of Mg.ATPγS. In contrast, Antarctic hairgrass α-Rca and Sitka spruce β-Rca formed stable hexamers under similar conditions. Spruce α-Rca exhibited unique redox-dependent oligomerization, forming large complexes stabilized by disulfide bonds. Thermal stability assays revealed significant nucleotide-induced stabilization in most isoforms, with hexamer formation enhancing stability and activity in select cases. Functional assays showed that hexamer-forming isoforms displayed superior Rubisco reactivation and ATP hydrolysis activities, even at low protein concentrations, while smaller oligomeric assemblies also supported activity in some species. These findings provide new insights into the structural and functional adaptations of Rca, highlighting the role of oligomeric assembly and environmental influences on its activity. This work lays a foundation for improving photosynthetic efficiency by targeting Rca isoforms tailored to specific environmental conditions.

**Supplementary Information:**

The online version contains supplementary material available at 10.1007/s00249-025-01794-4.

## Introduction

Rubisco (ribulose-1,5-bisphosphate carboxylase/oxygenase) activase (Rca) plays a key role in maintaining the catalytic activity of higher plant Rubisco during photosynthesis. Rubisco is one of the main limitations in the productivity of plants and is prone to inhibition by variety of sugar phosphates (Pearce 2006; Berry et al. 1987; Edmondson et al. 1990; Zhu and Jensen 1991; Gutteridge et al. 1986). Rubisco activase uses energy from ATP hydrolysis to remove these inhibitors, maintaining Rubisco activity. Recent studies have identified enzymes such as CA1P phosphatase that subsequently breakdown these inhibitory compounds (Andralojc et al. 2006; Bracher et al. 2015). Recent comprehensive reviews have further emphasized the structural complexity and regulatory flexibility of Rca, particularly in response to environmental cues and isoform-specific mechanisms (Bracher et al. 2015; Sparrow-Muñoz et al. 2023; Qu et al. 2023).

Through the activation of Rubisco, Rca is a key component in the regulation of photosynthetic processes in response to different conditions. Rca is inhibited by ADP, linking the activation state of Rubisco to the ADP/ATP ratio inside the chloroplast (Zhang and Portis 1999; Shen et al. 1991). Under light conditions, increasing ATP levels activate Rca, which then constitutively hydrolyzes ATP to maintain Rubisco activity. Rca activity is also regulated though two redox sensitive cysteine residues located in the C-terminal extension of the alpha-isoform and are subject to regulation by thioredoxin-f (Zhang and Portis 1999; Zhang et al. 2002). These cysteines are conserved within the α-isoform C-terminal extension, as shown in the alignment presented in Supplementary Fig. 1.

Most plants express two isoforms of Rubisco activase, with the longer alpha-isoform containing a 30-residue extension of the C-terminus compared to the shorter beta-isoform (Salvucci et al. 1987). These isoforms can be the result of alternative splicing (Werneke et al. 1989) or derived from separate genes (Salvucci et al. 2003). Some plants, including tobacco, only express the β-isoform. While studies of Arabidopsis Rca demonstrated that an equimolar ratio of the two isoforms is required to provide effective regulation in planta (Zhang et al. 2001), little has been done to quantify the ratio of the two isoforms *in planta*. Whereas spinach Rca appears to have an equal amount of the alpha- and beta-isoforms, soybean, kidney bean, pea, celery, oat, and barley appear to have a higher proportion of the beta-isoform relative to the alpha-isoform (Salvucci et al. 1987).

Unlike most other members of the AAA+ family of proteins, that tend to form hexamers in solution (Ogura and Wilkinson 2001; Erzberger and Berger 2006), the oligomeric state of Rubisco activase tends to be highly polydisperse. Previous studies of tobacco (Keown et al. 2013; Serban et al. 2018), cotton β-Rca (Kuriata et al. 2014; Chakraborty et al. (2013)), Arabidopsis β-Rca (Henderson et al. 2013), Oryza β- and α- Rca (Scafaro et al. 2016), and spinach β-Rca (Keown & Pearce 2014) have shown species ranging from monomers to 24 or more subunits, with the oligomeric state being strongly dependent on protein concentration. More recently, it was observed that spinach α-Rca exclusively forms hexamers in solution in the presence of Mg.ATPγS (Keown & Pearce 2014), as is observed for Arg-294 variants of tobacco Rca (Serben et al. 2018; Keown & Pearce 2014, Stotz et al. 2011, Blayney et al. 2011). Binding of ATP or ATPγS has been shown to increase the oligomeric state of spinach β-Rca (Keown & Pearce 2014) and cotton β-Rca (Kuriata et al. 2014), whereas similar studies of tobacco Rca showed little effect of nucleotide binding on oligomeric state (Keown & Pearce 2014).

Hexamer formation of spinach alpha-Rca or tobacco R294V Rca in the presence of ATPγS is associated with a substantial increase (>20°C) in thermal stability (Keown & Pearce 2014). The low thermal stability of Rca limits photosynthesis at high temperatures (Crafts-Brandner et al. 2000), leading to work aimed at improving photosynthesis through increasing the thermal stability of Rca (Kurek et al. 2007). Nucleotides such as ADP, ATP, and ATPγS have been shown to increase the thermal stability of Arabidopsis β-Rca, cotton β-Rca, tobacco Rca, and spinach β-Rca by up to 10 °C (Henderson et al. 2013; Kuriata et al. 2014; CraftsBrandner et al. 1997; Barta et al. 2010). Cotton Rca has similar thermal stability for both α- and β-isoforms, while the spinach α-isoform is more stable than the β-isoform (Keown & Pearce 2014).

Despite the importance of Rca in maintaining Rubisco activity, the nature of the interaction between Rca and Rubisco is still not clear. Many studies to date have focused on the CbbX protein, which functions as a Rubisco activase in prokaryotes and red algae and has ~50% sequence identity to higher plant Rca (Loganathan et al. 2016). To date, the best indication of the Rca-Rubisco interaction has been a detailed characterization of the interaction between Rubisco and CbbX from the photosynthetic bacteria *R. sphaeroides* (Bhat et al. 2017, Mueller-Cajar et al. 2011). In this case, CbbX forms a functionally active hexamer in the presence of ATP and RuBP, which docks side-on to the Rubisco hexadecamer and pulls the C-terminal end of Rubisco into the central pore, allowing release of the inhibitor. Studies of the red algal CbbX also suggest that it functions by drawing the C-terminus of Rubisco into the central pore (Loganathan et al. 2016).

While these studies have provided a clear insight into the mechanism by which higher plant Rubisco may be activated by Rubisco activase, there are some key differences between CbbX and higher plant Rca. Higher plant Rca has constitutive ATP hydrolysis activity, even in the absence of Rubisco, while CbbX Rca ATPase activity is stimulated by the presence of RuBP and Rubisco (Loganathan et al. 2016; Mueller-Cajar et al. 2011). Similarly, ATP and RuBP are required for the formation of active hexamers by *Rhodobacter sphaeroides* CbbX (Mueller-Cajar et al. 2011), while RuBP has not been reported to alter the oligomeric assembly of higher plant Rca. Unlike prokaryotic or higher plant Rca, CbbX from the red algae *Cyanidioschyzon merolae* functions as a heterooligomer that forms a hexamer in solution, even in the absence of any ligands (Loganathan et al. 2016).

Despite extensive studies on model species such as Arabidopsis, spinach, and tobacco, the diversity of Rca across phylogenetic groups remains underexplored. To address this gap, we investigate the Rca isoforms from four species adapted to distinct environmental conditions: cotton, creosote, Antarctic hairgrass, and Sitka spruce. An alignment of these species with the previously characterized tobacco and spinach enzymes is shown in Supplementary Fig. 1. Cotton and creosote are heat-adapted species, while Antarctic hairgrass and Sitka spruce thrive in colder climates. These species offer an unique opportunity to explore how evolutionary pressures have shaped the structural and functional properties of Rca. In this study, we characterize the oligomeric assembly, thermal stability, and enzymatic activity of α- and β-isoforms from these species. By examining their responses to nucleotides and redox conditions, we aim to elucidate the mechanisms underlying Rca function and adaptation. Understanding these mechanisms not only enhances our knowledge of photosynthetic regulation but also provides insights for improving crop productivity under diverse environmental conditions.

## Results and discussion

Many forms of Rca have been shown to form a continuous range of oligomeric species in solution, ranging from a single protomer up to large complexes consisting of >20 subunits. Following the observation that the α-isoform of spinach Rca forms thermally stable hexamers in the presence of ATPΥ[gamma]S (Keown & Pearce 2014), it was unclear whether this was a general trend among α-isoforms, or if hexamer formation was specific to spinach. To study this, α- and β-isoforms of cotton, creosote, Antarctic hairgrass, and spruce Rca were purified and tested for their propensity to form hexamers in solution using analytical ultracentrifugation. An alignment of these protein sequences is shown in Fig S1, while a phylogenetic tree is shown in Fig S2.

**Fig. 1:**
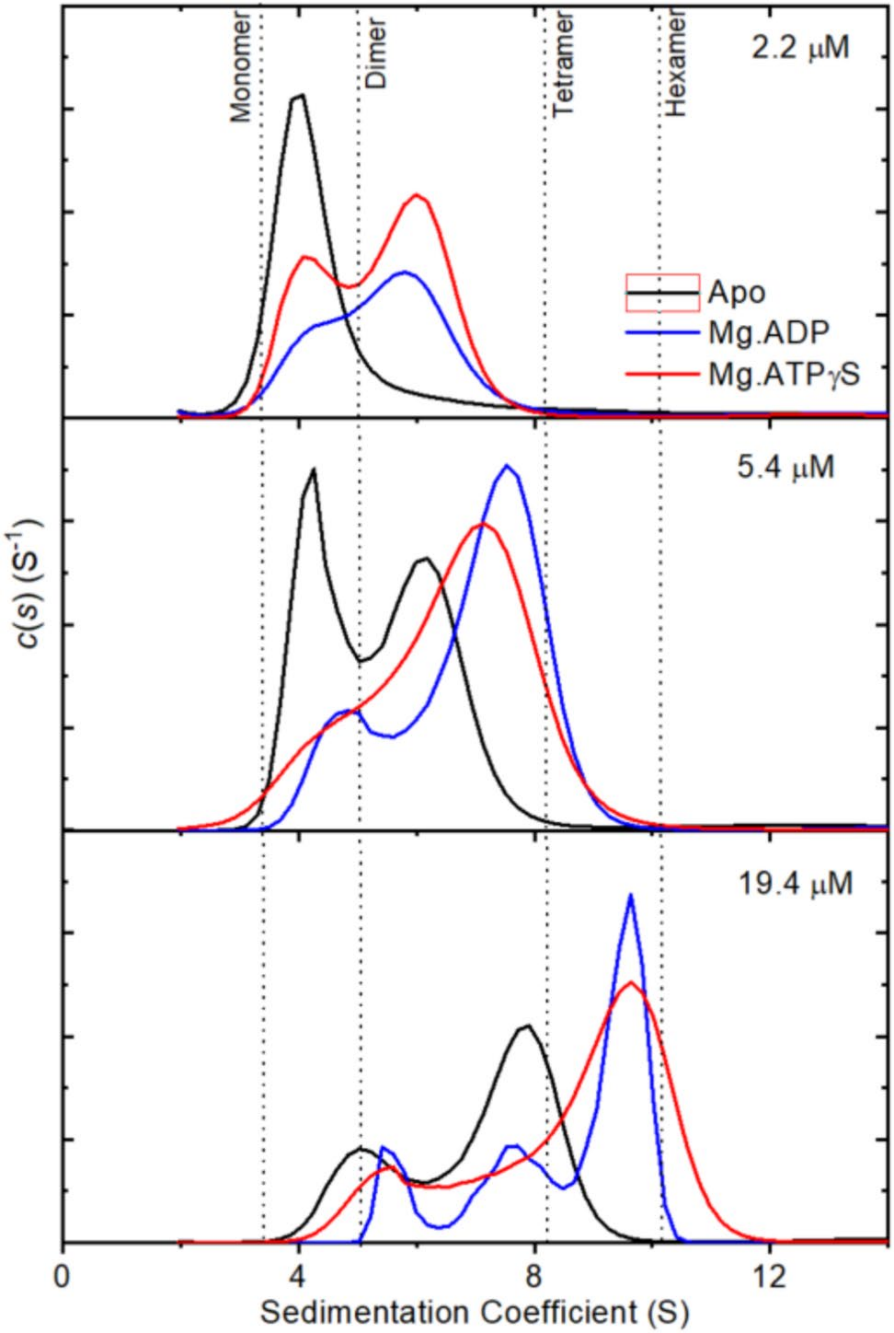
Continuous size distributions of creosote α-Rca determined by analytical ultracentrifugation. Sedimentation velocity experiments were carried out at different Rca concentrations in the absence of Ligand or in the presence of 0.2 mM Mg.ADP or Mg.ATPγS. Data were fitted to a sedimentation coefficient distributions c(s) model. For clarity, data are shown for three of the six protein concentrations tested. Vertical dotted lines indicate the expected sedimentation coefficient of different oligomeric species

Analytical ultracentrifugation is a powerful technique for measuring the oligomeric state of proteins and has been used previously to characterize tobacco and spinach Rca. This technique has advantages over other methods in that proteins do not need to be labeled, the proteins are in solution, rather than as crystals or in gas phase, and the exact protein and ligand concentrations are known for the system. In this case, as previously, sedimentation coefficient distributions c(s) have been fitted, which provide an indication of species in solution. For heterogeneous systems such as Rca, the appearance of peaks in these distributions is governed by the kinetics of the interaction and may occur at positions that do not necessarily represent the sedimentation coefficient (Dam et al. 2005). To accurately compare different Rca isoforms, species, and the effect of ligands, the c(s) has been integrated to provide a weight averaged S value (Dam & Schuck 2004). Figure 1 shows an example of this for the α-isoform of creosote Rca.

### Cotton and creosote Rubisco Rca do not appear to form discrete hexamers in solution

It has previously been shown that the β-isoform of cotton Rca forms a range of oligomeric species in solution (Kuriata et al. 2014; Chakraborty et al. 2013); however, little is known about the assembly of the α-isoform. Fluorescence correlation spectroscopy studies have suggested that cotton β-Rca exists as a mixture of various oligomeric states in solution, including monomers, dimers, tetramers, hexamers, and 24-mers (Chakraborty et al. 2013).

**Fig. 2:**
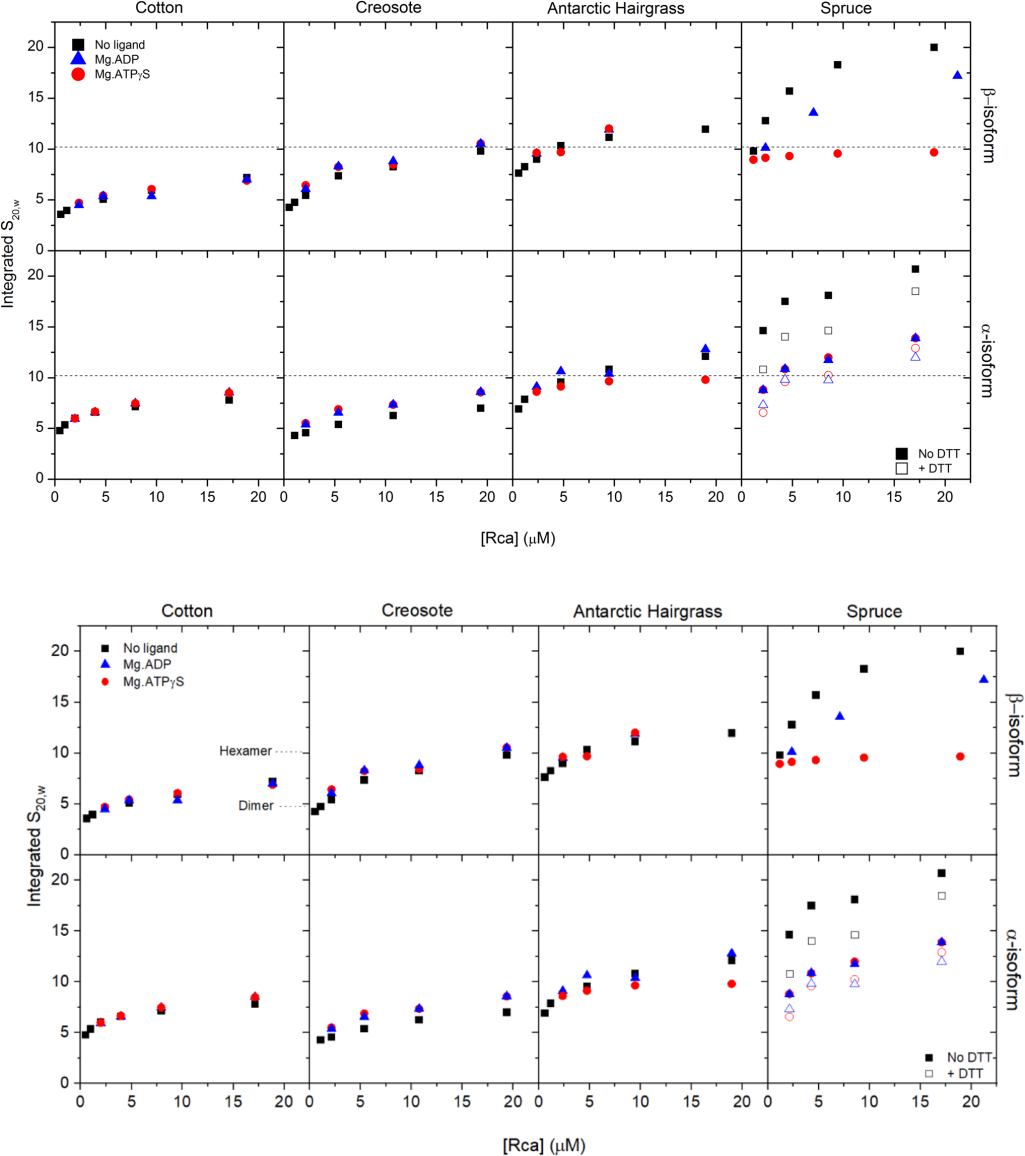
Dependence of Rca oligomeric size on protein concentration. Sedimentation velocity experiments were carried out at different Rca concentrations in the absence of nucleotide (squares), or in the presence of 0.2 mM Mg.ATPγS (circles) or Mg.ADP (triangles). The integrated sedimentation coefficient was calculated for each run, and converted to *s*_20,w_ values (right axis)

Our current studies using analytical ultracentrifugation show that both isoforms of cotton Rca form highly polydisperse complexes in solution, and the size of these complexes is dependent on protein concentration (Fig 2), as has been seen previously for cotton β-Rca (Kuriata et al. 2014; Chakraborty et al. 2013). At concentrations below 1 μM, the β-isoform forms very small species with a weight averaged sedimentation coefficient of 3.6S at 0.6 μM close to that expected for a monomer (predicted S of 3.1), as has been observed in other studies (Kuriata et al. 2014; Chakraborty et al. 2013). At 2.3 μM, the average sedimentation coefficient is similar to that expected for a dimer, and at 10 μM, the enzyme has an average sedimentation coefficient similar to a trimer. The α-isoform of cotton Rca tends to form slightly larger species than the β-isoform, with an average sedimentation coefficient corresponding to dimeric species predominating at 0.5 μM, trimers at 1.2 μM and species larger than hexamers above 5 μM (Fig 2). The addition of Mg.ADP or Mg.ATPγS did not change the oligomeric state of the β- or α-isoforms, and unlike previous observations of spinach α-Rca (Keown & Pearce 2014; Stotz et al. 2011), cotton α-Rca did not form discrete hexameric species upon addition of Mg.ATPγS.

It is not clear why the current study does not agree with previous fluorescence correlation spectroscopy studies, in which the dissociation constant for hexamer formation of cotton β-Rca in the presence of Mg.ATPγS was calculated to be tenfold lower than in the presence of Mg.ADP, with hexamers predicted to comprise 60–80% of Rca subunits between 8 and 70 µM protein (Kuriata et al. 2014). The weight averaged sedimentation coefficient of cotton β-Rca at 20 µM was 7S, which equates to 3–4 subunits. The distance distribution plot showed few species larger than 10S, the expected sedimentation coefficient for a hexameric assembly. The addition of 5 mM MgCl_2_ did not significantly alter the distribution, and neither did the addition of 0.5 mM ADP or 0.5 mM ATPγS.

Although the activity and thermal stability of creosote Rca have previously been studied (Salvucci & CraftsBrandner 2004), and structural information is known for the C-terminal domain of creosote β-Rca (Henderson et al. 2011), the oligomeric nature of these enzymes was unknown. Like tobacco and cotton Rca enzymes, creosote α- and β-Rca formed polydisperse mixtures in solution (Fig 1), and the size of these oligomers was dependent on protein concentration (Figs. 1 and 2). The size of these complexes was similar to those observed for cotton Rca, with creosote β-Rca ranging from monomer/dimers at 0.6 μM to hexameric at 20 μM, and creosote α-Rca forming smaller species, ranging from monomer/dimers at 1.2 μM to trimer/tetramers at 20 μM. Addition of Mg.ADP or Mg.ATPγS tended to increase the size of the oligomers in solution, as seen by analytical ultracentrifugation, with the α-isoform increasing from 7.0 S to 8.6 S at 20 μM in the presence of either nucleotide, and the β-isoform increasing from 9.8 S to 10.6 S.

### Antarctic hairgrass α-Rca forms discrete hexamers in the presence of Mg.ATPγS

To examine whether Rca enzymes from species that grow in contrasting environments have different oligomeric assemblies, the α- and β-isoforms of Antarctic hairgrass Rca were also studied. While tobacco, cotton, and creosote are adapted to grow at high temperatures, Antarctic hairgrass and spinach are endemic to colder regions (Salvucci & CraftsBrandner 2004).

In the absence of nucleotide, both isoforms of Antarctic hairgrass formed polydisperse complexes in solution, with less concentration-dependent changes in size compared to Rca from other species (Fig 2). At low protein concentrations (0.5 µM, the lowest protein concentration that was able to be measured by analytical ultracentrifugation), Antarctic hairgrass Rca formed complexes with a weight averaged sedimentation coefficient of 6.9 S and 7.6 S for the α- and β-isoforms (an average size of 3–4 subunits). These complexes are larger than the complexes formed by cotton, creosote, or tobacco Rca at equivalent concentrations.

**Fig. 3:**
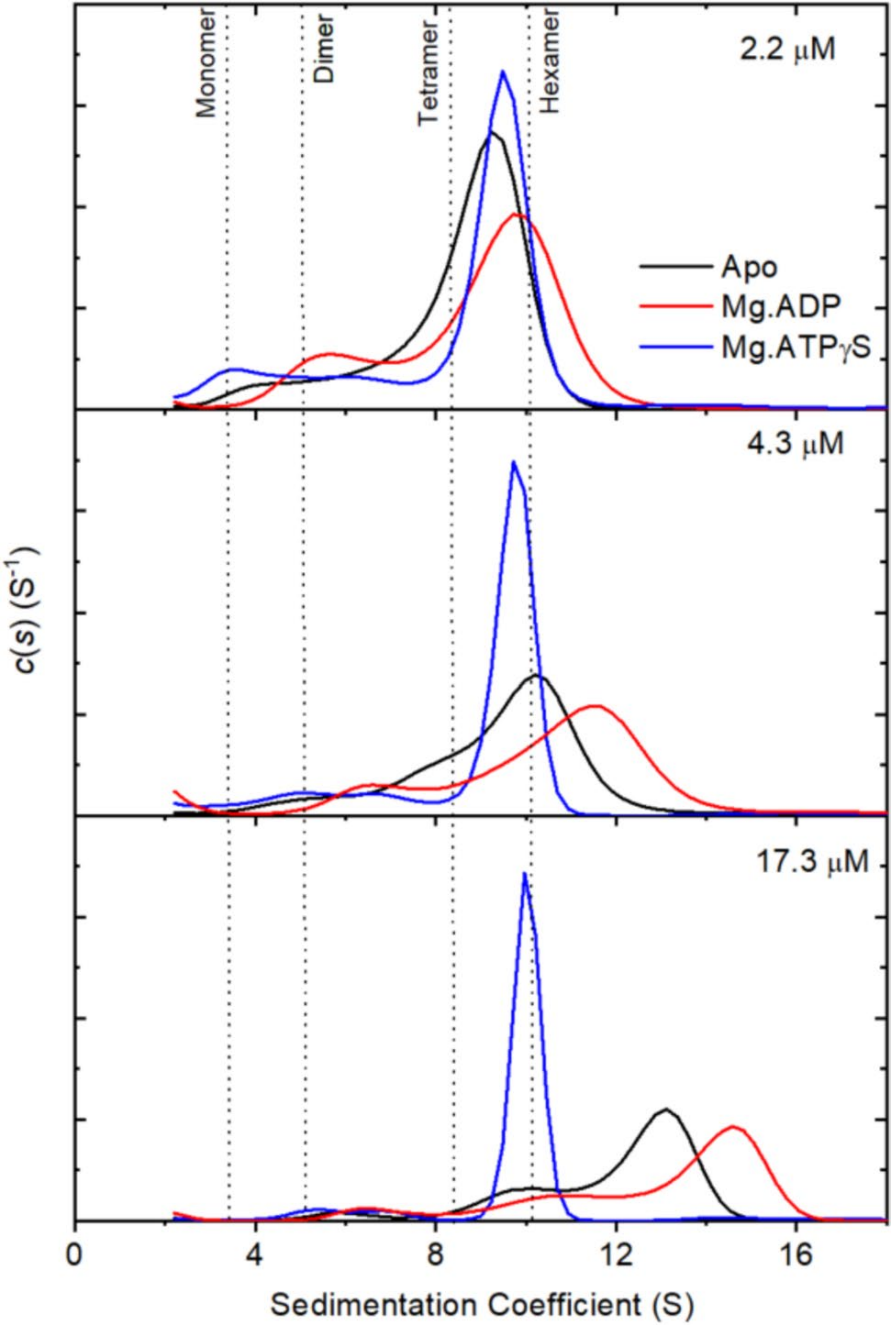
Continuous size distribution of Antarctic hairgrass α-Rca determined by analytical ultra-centrifugation. Sedimentation velocity experiments were carried out at different protein concentrations in the absence of ligand or in the presence of Mg.ADP or ATPγS. Data were fitted to a sedimentation coefficient distributions c(s) model. For clarity, data are shown for three of the six protein concentrations tested. Vertical dotted lines indicate the expected sedimentation coefficient of different oligomeric species

Addition of Mg.ATPγS to the α-isoform of Antarctic hairgrass Rca resulted in the formation of monodisperse species at all protein concentrations measured (Fig 3), as has previously been observed for spinach α-Rca (Keown & Pearce 2014). The size of this species is consistent with that predicted for a hexamer (10.2 S), and the peak is narrower relative to that observed under other conditions, suggesting that the species forms a tight complex. The addition of Mg.ATPγS resulted in little change in oligomeric state of the β-isoform of Antarctic hairgrass Rca (Fig 2), and there was still a polydisperse distribution of species in solution. The presence of Mg.ADP resulted in a small increase in the oligomeric state of both α- and β-isoforms of Antarctic hairgrass Rca, though the distribution remained polydisperse.

### Sitka spruce β-Rca forms discrete hexamers in the presence of Mg.ATPγS

To study phylogenetically diverse Rubisco activase enzymes, genes encoding the putative α- and β-isoforms of Sitka spruce (*Picea sitchensis)* were synthesized, cloned into an expression plasmid, and expressed in *E. coli*. Unlike spinach, tobacco, creosote, and cotton Rca, both isoforms of spruce Rca migrated as apparent dimers under non-reducing SDS-PAGE conditions but as monomers when dithiothreitol was included, indicating the presence of an intersubunit disulfide bond (Fig S3). As SDS-PAGE disrupts non-covalent interactions but retains covalent linkages, this observation suggests that the dimeric forms are covalently linked via disulfide bridges (data not shown).

In the absence of nucleotide, both spruce Rca isoforms exist as very large species. At 2 μM, spruce α-Rca has a weight averaged sedimentation coefficient of 15 S, which corresponds to >10 subunits, and spruce β-Rca has a sedimentation coefficient of 13 S, which corresponds to an average size of >8 subunits. This is larger than the complexes formed by Rca from other species at an equivalent concentration. The unusually large complexes formed by spruce α-Rca, even in the absence of nucleotide, raise the possibility that these could represent fibrillar or aggregated assemblies, similar to those previously reported for tobacco Rca at high concentrations (Serban et al. 2018). These forms are enzymatically inactive (Serban et al. 2018), suggesting that some high-order assemblies may represent non-functional or aggregated states. Although our sedimentation data support a large, polydisperse population, further work such as electron microscopy or light scattering would be required to confirm whether these higher order structures represent ordered fibrils or dynamic oligomers.

Inclusion of Mg.ATPγS induces the formation of discrete hexamers by spruce β-Rca at all protein concentrations tested (1–10 μM), as indicated by a single narrow peak in the size distribution plot with a sedimentation coefficient of ~9.5 S. This is slightly smaller than is seen for spinach α-Rca and Antarctic hairgrass α-Rca, due to the smaller size of the β-isoform compared to the α-isoform. While the presence of Mg.ADP reduced the size of spruce β-Rca oligomers compared to no ligand, there was still a polydisperse distribution. Addition of Mg.ADP or Mg.ATPγS to spruce α-Rca reduced the oligomeric size to a similar extent, with a polydisperse distribution.

Previous work has shown that the α-isoform of Arabidopsis Rca contains two cysteine residues at the C-terminus that are activated by thioredoxin-f or dithiothreitol (Zhang and Portis 1999). Following the observation that Sitka spruce Rca isoforms appear to form an intersubunit disulfide bond, 5 mM dithiothreitol was included to test whether changes in redox conditions changed the oligomeric state. Under reducing conditions, the oligomeric assembly of spruce α-isoform was decreased, suggesting that the presence of the disulfide bond promotes oligomerization. Addition of DTT caused no change in oligomer distribution for any tobacco, spinach, cotton, or creosote isoforms, or for the β-isoform of spruce Rca (data not shown).

### Rubisco reactivation and ATP hydrolysis activity is correlated to oligomeric state

While most AAA+ proteins function as a hexamer (Erzberger and Berger 2006), there are several instances where enzymes form functional dimers that have ATP hydrolysis activity (Maisel et al. 2012; Bochman et al. 2008; Monroe et al. 2014). Hydrolysis of ATP by Rca requires a critical concentration of protein, corresponding to the minimum oligomeric state necessary for activity. Previous studies of tobacco and spinach β-Rca suggested that the minimum oligomeric state required for ATP hydrolysis and Rubisco reactivation was 2–4 subunits (Keown et al. 2013; Stotz et al. 2011; Keown & Pearce 2014). Studies of *Oryza* β- and α-Rca also showed that ATPase and Rubisco reactivation activity were dependent on protein concentration (Scafaro et al. 2016).

**Fig. 4:**
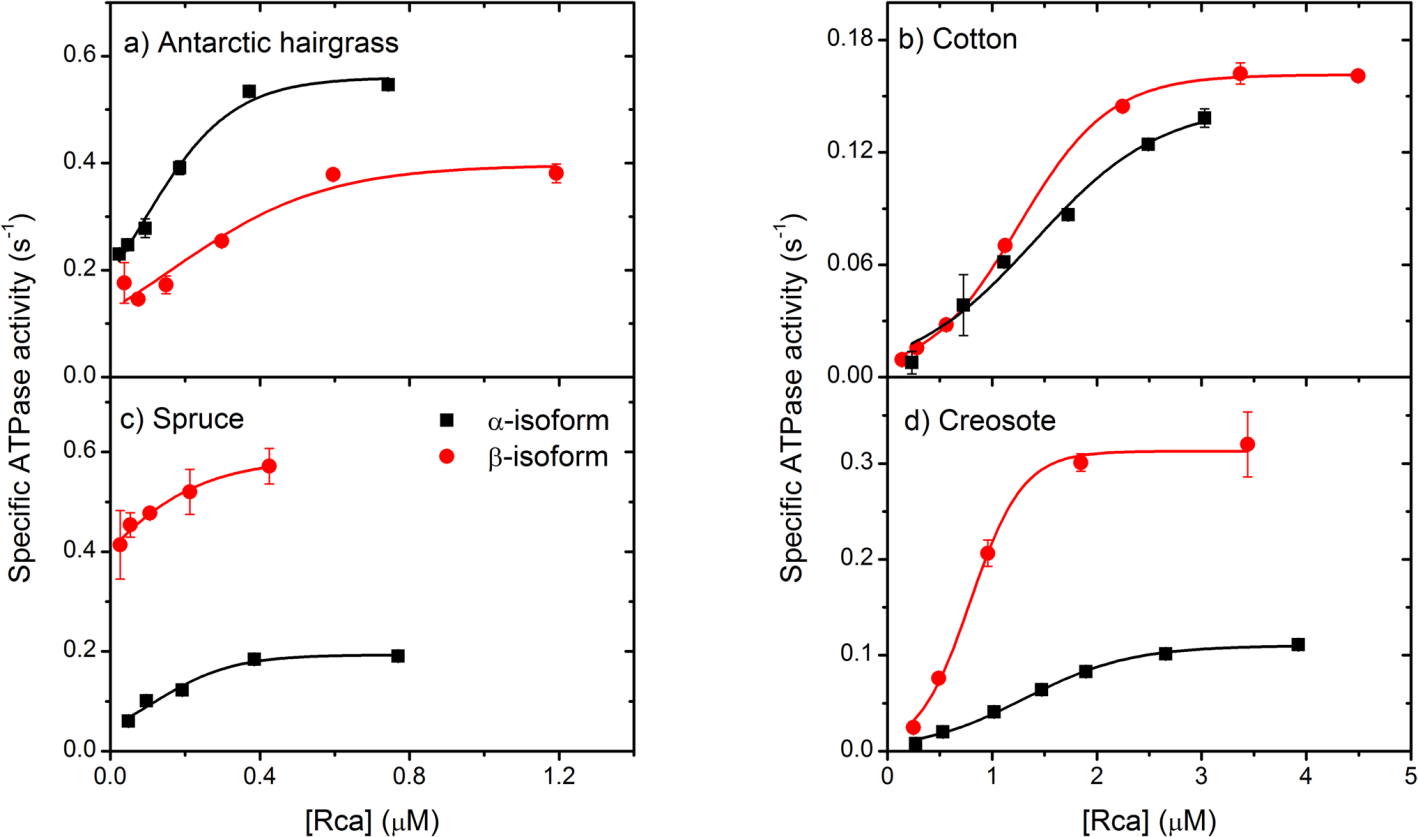
Dependence of ATPase activity on Rca concentration. Specific ATPase activity was measured for a range of protein concentrations using a coupled assay. Error bars represent the S.D. of three replicates, and are not visible due for some values due to small variance

**Fig. 5:**
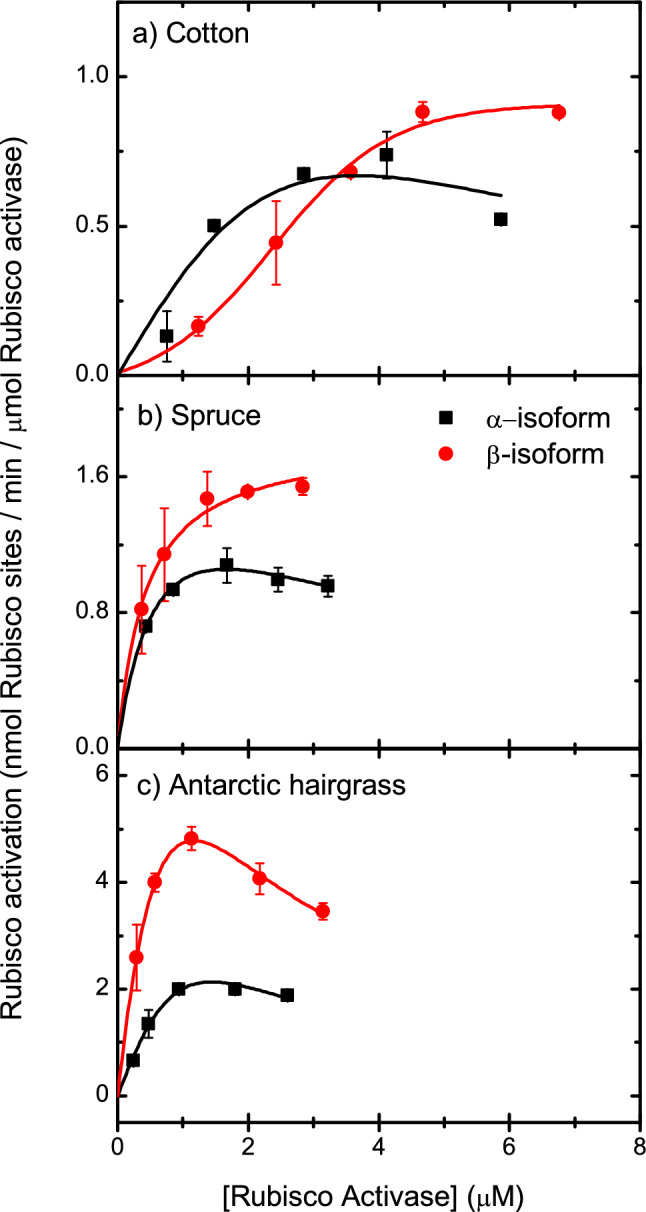
Dependence of Rubisco reactivation activity on Rca concentration. Ability of Rca to activate spinach ER was measured for a range of protein concentrations using a coupled assay. Error bars represent the S.D. of three replicates, and are not visible due to small variance in some values

All isoforms of Rca showed a decrease in activity at low protein concentrations (Figs. 4 and 5). Rubisco reactivation assays were carried out as previously described, and the ER form of spinach Rubisco was used as a substrate for all reactivation assays due to lack of availability of all the native corresponding Rubisco enzymes. A sigmoidal or hyperbolic function was fitted to the data to determine the protein concentration at which the activity was 50% of the maximal rate.

Cotton α- and β-isoforms had 50% maximal ATPase activity at 1.4 and 1.3 μM, respectively, and 50% maximal Rubisco activation activity at 0.7 and 0.9 μM, respectively (Figs. 4 and 5). From analytical ultracentrifugation data, cotton α-Rca has a sedimentation coefficient of 5.3 S at 1.0 μM and β-Rca, which formed smaller species, has a sedimentation coefficient of 4.0 S at 1.2 μM. Similarly, creosote α- and β-isoforms had 50% maximal ATPase activity at 1.3 and 0.73 μM, respectively, which correspond to a sedimentation coefficient of 4.4 S at 1.2 μM and 0.6 μM. These sedimentation coefficients correspond closely to the expected sedimentation coefficient of a dimer (4.9S) and are consistent with full activity being observed at protein concentrations less than required for formation of larger (> 6 subunits) species.

In the current study, neither α- nor β-isoforms of creosote Rca were able to reactivate the ER form of spinach or tobacco Rubisco (data not shown). It has previously been shown that Solanaceae Rca activates only Solanaceae Rubisco, and that non-Solanaceae Rubisco is generally activated by any non-Solanaceae plant or green algal Rca (Wang et al. 1992), but this has not been extensively studied. It has been shown that Rca interacts with Rubisco via the sensor 2 domain of Rca associating with the βC-βD loop of the Rubisco large submit (Li et al. 2006). More recently, it has been suggested that the α4-β4 loop of Rca is also important in interacting with Rubisco (Shivhare & Mueller-Cajar 2017). This suggests that there may be sequence differences that prevent the Creosote Rca from binding to spinach or tobacco Rubisco.

Antarctic hairgrass Rca, which appears to form much larger species in solution than creosote or cotton Rca, maintained activity down to low protein concentrations, having 50% Rubisco reactivation activity at 0.02 and 0.4 μM and 50% ATPase activity at 0.078 and 0.15 μM for the α- and β-isoforms (Figs. 4 and 5). These concentrations are below the range of detection for analytical ultracentrifugation, and the trend is like that observed for spinach α-Rca and tobacco R294V Rca, which form closed hexamers in solution. It is likely that the hexameric species observed by analytical ultracentrifugation for the α-isoform of Antarctic hairgrass Rca are stable down to very low protein concentrations.

Consistent with the observation that spruce β-Rca forms discreet hexamers in solution, Rubisco reactivation and ATP hydrolysis activity were maintained at very low protein concentrations, with 50% of maximal activity estimated to occur at 0.01 μM (Figs. 4 and 5). The large complexes observed for spruce α-Rca also maintained activity at low protein concentrations, with 50% Rubisco reactivation activity at 0.17 μM and 50% maximal ATPase activity observed at 0.12 μM. Inclusion of DTT did not alter the Rubisco reactivation or ATP hydrolysis activity of either spruce Rca isoform, suggesting that the redox state is not necessarily important for activity.

Together, these results are consistent with the previous work with tobacco Rca (Keown et al. 2013) and more recent studies (Serban et al. 2018), which show that Rca activity can be maintained by smaller or dynamically assembling oligomers. These studies demonstrate that subunit exchange and dynamic oligomerization are critical for maintaining active assemblies, challenging the assumption that a static hexamer is required for function. Neither isoform of cotton nor creosote Rca was observed to form discrete hexameric structures in solution, and full Rubisco reactivation and ATPase activity were observed at concentrations lower than that required for much of the enzyme to be hexameric or larger. Several members of the AAA+ family, such as clamp loader and initiator proteins (Erzberger et al. 2006; Bowman et al. 2004), form spiral assemblies in solution, and Rca may function as an open spiraling structure rather than a closed hexamer. The case for a spiraling assembly is also supported by the crystal forms found for both tobacco (Stotz et al. 2011) and Arabidopsis Rca enzymes (Hasse et al. 2015), which both crystallized as spiraling oligomers with P6 symmetry.

As has previously been reported for spinach Rca (Keown & Pearce 2014), the α-isoform of Antarctic hairgrass Rca and the β-isoform of spruce Rca were able to form hexamers in solution in the presence of Mg.ATPγS and is likely to function as a hexamer *in vivo*. While genes for both Rca isoforms are available, the relative abundance of each isoform in planta is not known.

While our current assays did not show any change in oligomeric state in the presence of Rubisco or RuBP (data not shown), the substrate of Rubisco, it remains possible that Rubisco itself could influence Rca oligomeric assembly *in vivo*. In prokaryotic systems, such as with CbbX, Rubisco promotes the formation of active hexamers (Bhat et al. 2017; Mueller-Cajar et al. 2011). Although such stimulation has not been observed for higher plant Rca, future studies will be needed to determine whether Rubisco interaction alters the equilibrium of oligomeric states or stabilizes specific assemblies under physiological conditions.

### Thermal stability is similar for most Rca isoforms

Many studies have shown that binding of nucleotides to Rca increases their thermal stability (Salvucci et al. 2003; Kuriata et al. 2014; Henderson et al. 2013; CraftsBrandner et al. 1997; Barta et al. 2010). This effect is most profound for spinach α-Rca and tobacco R294V Rca, in which binding of ATPγS is associated with an increase in thermal stability of >20°C (Henderson et al. 2013). For both the enzymes, binding of ATPγS is also associated with the formation of discrete hexamers, suggesting that hexamer formation greatly increases thermal stability (Keown & Pearce 2014). Changes in protein stability induced by ligand binding correlate with changes in protein flexibility (Celej et al. 2003), and in this case, it is likely that binding of ATPγS reduces the flexibility of Rca, which is thought to have extended the N- and C-terminal domains (Keown & Pearce 2014; Stotz et al. 2011). The exact change in Rca conformation that occurs upon ADP or ATP binding is unknown, but it is thought to involve movement of the small domain relative to the large domain, based on changes in the intrinsic fluorescence (Wang et al. 1993; Wang & Portis 2006).

**Fig. 6:**
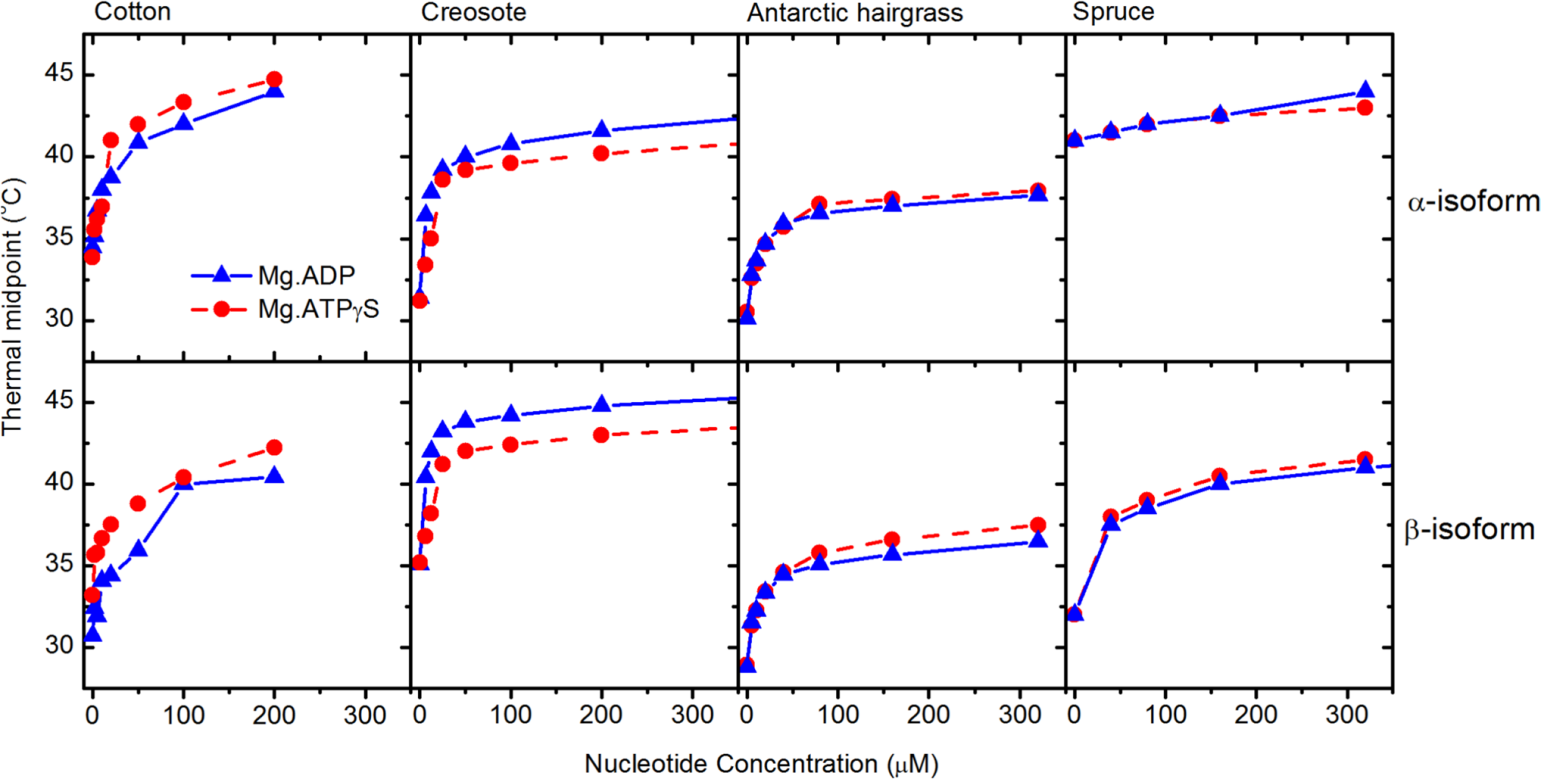
Dependence of thermal stability on nucleotide concentration. Thermal stability was measured for a range of nucleotide concentrations for different Rca enzymes (0.2 mg/mL) using thermal shift assays. Experiments were carried out at varying concentrations of Mg.ATPγS (triangles) or Mg.ADP (circles). Values shown are the average of ≥3 replicates. Replicates showed consistent values

The thermal stability of spruce β-Rca and Antarctic hairgrass α-Rca, which form discreet hexamers in the presence of ATPγS, showed a similar response for the binding of ADP and ATPγS, with an increase in stability of 12 °C (Fig 6). This suggests that the formation of hexameric species alone is not sufficient to provide an extensive increase in thermal stability.

Both isoforms of cotton, creosote, and Antarctic hairgrass showed a similar response to nucleotide binding, with an increase in stability of 7–12 °C (Fig 6), which is comparable to that previously observed for cotton Rca (Salvucci et al. 2003). The increase in stability was similar for the binding of both Mg.ATPγS and Mg.ADP. For most isoforms, a nucleotide concentration of 10–60 μM was required for 50% of the maximum thermal stability. It is interesting to note that the Rca enzymes from Antarctic hairgrass, which grow naturally at cold temperatures, had similar thermal stability to Rca from both cotton and creosote, which grow naturally at very warm temperatures.

In contrast, the thermal stability of spruce α-Rca was not substantially altered by the presence of nucleotide, with an increase in thermal stability of 2–3 °C even in the presence of 320 µM nucleotide (Fig 6). This isoform tended to form larger complexes in solution than the other variants tested, with species larger than 9 S at all concentrations tested. Previous work on spinach and tobacco Rca showed that higher order oligomers have higher thermal stability than smaller species (Keown & Pearce 2014), and it is likely to be the case that the large oligomers formed by spruce α-Rca have high heat stability.

## Conclusion

Rubisco activase (Rca) is indispensable for maintaining photosynthetic efficiency by restoring Rubisco activity under diverse physiological and environmental conditions. This study provides a comparative analysis of α- and β-isoforms of Rca from species adapted to contrasting thermal environments, including cotton, creosote, Antarctic hairgrass, and Sitka spruce. Our findings highlight the significant diversity in the oligomeric assembly, thermal stability, and functional activity of Rca isoforms, revealing species-specific adaptations that reflect their ecological niches.

Unlike typical AAA+ proteins, which predominantly form hexamers, Rca exhibits a wide range of oligomeric states (Keown et al. 2013; Serban et al. 2018; Kuriata et al. 2014; Henderson et al. 2013; Scafaro et al. 2016; Keown and Pearce 2014; Stotz et al. 2011; Chakraborty et al. 2013; Chakroborty et al. 2012). Cotton and creosote isoforms displayed highly polydisperse assemblies without forming discrete hexamers, even in the presence of Mg.ATPγS, suggesting that functional activity in these species may be mediated by smaller oligomeric units. Conversely, Antarctic hairgrass α-Rca and Sitka spruce β-Rca formed stable hexamers under similar conditions, linking hexamer formation to enhanced activity and thermal stability. These findings emphasize the influence of environmental selection pressures on the structural properties of Rca. It has recently been proposed that changes in Rca oligomeric structure could play a role in Rubisco regulation (Peterson Forbrook et al. 2017; Hazra et al. 2015), with faster subunit exchange when ADP was bound and slower exchange when bound to ATP or ATPγS

Thermal stability assays showed that nucleotide binding universally improved stability, though the extent varied between isoforms. The formation of stable hexamers was associated with greater stability in Antarctic hairgrass α-Rca and Sitka spruce β-Rca, while larger polydisperse assemblies in spruce α-Rca appeared inherently stable, even without nucleotide binding. These results indicate that hexamer formation and oligomeric diversity are key determinants of Rca’s functional resilience, particularly under temperature extremes.

Functionally, the ability of Rca isoforms to hydrolyze ATP and reactivate Rubisco was closely tied to their oligomeric states and protein concentration. In the data presented here, and previously for other species (Keown et al. 2013; Keown and Pearce 2014; Scafaro et al. 2016), Rca is able to activate Rubisco and hydrolyze ATP at low protein concentrations. Hexamer-forming isoforms demonstrated robust activity even at low protein concentrations, whereas isoforms forming smaller or polydisperse oligomers required higher concentrations to achieve full activity. The protein concentration in the chloroplast is very high (~400 mg/ml), with a Rubisco concentration of ~200–250 mg/ml (similar to the concentration in protein crystals) and an Rca concentration of ~20 mg/ml (Süss et al. 1996; Yamori and von Caemmerer 2009). The exact arrangement of Rubisco and Rubisco activase in the chloroplast is unclear, but immunoelectron microscopy has shown that both are evenly distributed around the stroma (Süss et al. 1996). These findings suggest that while hexamer formation enhances efficiency in some isoforms, smaller oligomers may provide functional flexibility, potentially enabling Rca to adapt to the high-protein, gel-like environment (Wise et al. 2006) of the chloroplast stroma. Such dynamic behavior, including rapid subunit exchange and transient complex formation, has been proposed as a core mechanism in Rca function (Serban et al. 2018), and may explain how polydisperse species in cotton or creosote Rca retain activity at physiologically relevant concentrations.

Recently, there has been renewed interest in surveying the catalytic properties of Rubisco from a range of diverse species, to appreciate the variability in enzyme function. These studies have included diatoms (Young et al. 2016), wheat (Prins et al. 2016), as well as a range of terrestrial plants (Galmes et al. 2014). As stated in a recent commentary by Hansen (Hanson 2016), “These assays should be conducted using different combinations of large and small subunits and paired with measures of activation by multiple forms of Rubisco activase.”

The results presented here broaden our understanding of the structural and functional diversity of Rca, providing insights into how these enzymes are adapted to specific environmental conditions. The observed differences between isoforms and species underscore the importance of evolutionary pressures in shaping Rca’s properties. This knowledge has significant implications for efforts to engineer crops with enhanced photosynthetic performance and resilience to environmental stress. Future work could explore the interplay between Rca isoform expression, Rubisco activation dynamics, and overall photosynthetic efficiency in planta, paving the way for targeted biotechnological interventions.

## Experimental procedures

### Protein expression, purification, and activity

Plasmids encoding the α- and β-isoforms of cotton, creosote, and Antarctic hairgrass Rca were the kind gift of Mike Salvucci. Amino acid sequences for *Picea sitchensis* Rca were determined from NCBI (accession ABK24548 and ABK25255). Chloroplast transit peptide regions were located using ChloroP and were removed from the construct. Genes were synthesized by Epoch Life Science Inc (Missouri City, TX 77496) and cloned into the pET11a vector. Protein expression and purification for all Rca enzymes were carried out as described previously (Ogura and Wilkinson 2001; Henderson et al. 2013). ATPase activity and Rubisco reactivation assays were carried out using a continuous coupled assay, as previously described (Wang et al. 1992). Rubisco reactivation assays used tobacco or spinach Rubisco as described in the text.

### Protein characterization

Experiments were carried in buffer containing 20 mM Bis-Tris propane (1,3-bis[tris(hydroxymethyl)methylamino]propane), pH8.0, 20 mM KCl and 0.2 mM EDTA buffer. 5 mM MgCl_2_ was included where nucleotide has been included, and the nucleotide concentration was 0.2 mM, unless otherwise indicated. Analytical ultracentrifugation experiments were carried out at 20 °C for all variants of the enzyme, using conditions described previously (Keown et al. 2013). Briefly, proteins were centrifuged at various speeds (typically 45,000 rpm) and radial absorbance data were collected at appropriate wavelengths (typically 280nm). Data were fitted to a continuous size distribution (cs) model using the program SEDFIT and weight average sedimentation coefficients were calculated for the fits. Where indicated, 0.2 mM ADP or ATPγS was included in the buffer. Thermal stability was measured as previously described (Serban et al. 2018; Prins et al. 2016), using a protein concentration of 10–20 µM.

## Supplementary Information

Below is the link to the electronic supplementary material.

Supplementary file1 (DOCX 174 KB)

## Data Availability

All analytical ultracentrifugation, thermal stability and kinetic data generated in this study are available from the authors without restriction upon request.
