# Supplementary material for: Ribulose-1,5-bisphosphate carboxylase/oxygenase activase isoforms from diverse species show differences in oligomeric structure, thermal stability, and activity
Source: Eur Biophys J. 2025 Sep 11;54(6):403–14. doi: 10.1007/s00249-025-01794-4 (PMC12552412; doi:10.1007/s00249-025-01794-4)
Supplement: Supplementary file 1 — Supplementary file1 (DOCX 174 KB) [file 249_2025_1794_MOESM1_ESM.docx]

Spinach -----AAENEEKNTDKWAHLAKDFSDDQLDIRRGKGMVDSLFQAPADAGTHVPIQSSFEY

Picea ---AEIDEGKQTDKDKWKGLAFDESDDQMDIRRGKGKVDSLFQAPMGSGTHNVVMSTYDY

Tobacco AEQIDVDPKKQTDSDRWKGLVQDFSDDQQDITRGKGMVDSLFQAPTGTGTHHAVLQSYEY

Hairgrass -MAKDIDEGKQTDGDKWKGLAYDISDDQQDITRGKGIVDSLFQAPMGDGTHEAVLSSYEY

Cotton AAEKEIDEETQTEKDRWKGLAYDISDDQQDITRGKGMVDSLFQAPMNDGTHYAVMSSYEY

Creosote --AQEISEDQQTDKDKWKGLAYDISDDQQDITRGKGMVDTLFQAPMQSGTHYAVMSSYDY

:.: *:* *. * **** ** **** **:***** *** : .:::*

Spinach ESQGLRKYDIDNMLGDLYIAPAFMDKLVVHITKNFLNLPNIKIPLILGVWGGKGQGKSFQ

Picea ISTAQRTYDFDNTMDGYYIAPSFMDKLLVHISKNFMNLPNIKVPLILGIWGGKGQGKSFQ

Tobacco VSQGLRQYNLDNKLDGFYIAPAFMDKLVVHITKNFLKLPNIKVPLILGIWGGKGQGKSFQ

Hairgrass VSQGLKKYDFDNTMGGFYIAPAFMDKLVVHLSKNFMTLPNIKIPLILGIWGGKGQGKSFQ

Cotton ISQGLKTYNLDNNMDGFYIAPAFMDKLVVHISKNFMSLPNIKVPLILGIWGGKGQGKSFQ

Creosote ISQGLRQYNLDNNMDGFYIAPAFMDKLVVHITKNFLSLPNIKIPLILGIWGGKGQGKSFQ

* . : *::** : ****:*****:**::***:.*****:*****:***********

Spinach CELVFAKLGINPIMMSAGELESGNAGEPAKLIRQRYREAADLIAKGKMCALFINDLEPGA

Picea CELVFAKLGINPIMMSAGELESGDAGEPAKLLRKRYREASDIVKKGKMCVLFINDLDAGA

Tobacco CELVFRKMGINPIMMSAGELESGNAGEPAKLIRQRYREAAEIIRKGNMCCLFINDLDAGA

Hairgrass CELVFAKMGINPIMMSAGELESGNAGEPAKLIRQRYREAADMIKKGKMCCLFINDLDAGA

Cotton CELVFAKMGINPIMMSAGELESGNAGEPAKLIRQRYREAADIIKKGKMCALFINDLDAGA

Creosote CELVFAKMGINPIMMSAGELESGNAGEPAKLIRQRYREAADIIKKGKMCCLFINDLDAGA

***** *:***************:*******:*:*****:::: **:** ******: **

Spinach GRMGGTTQYTVNNQMVNATLLNIADNPTNVQLPGMYNKQDNARVPIIVTGNDFSTLYAPL

Picea GRMGSTTQYTVNNQMVNATLMNIADNPTNVQLPGMYNKQDNPRVPIVVTGNDFSTLYAPL

Tobacco GRMGGTTQYTVNNQMVNATLMNIADNPTNVQLPGMYNKQENARVPIIVTGNDFSTLYAPL

Hairgrass GRMGGTTQYTVNNQMVNATLMNIADAPTNVQLPGMYNKEENPRVPIXVTGNDFSTLYAPL

Cotton GRMGGTTQYTVNNQMVNATLMNIADNPTNVQLPGMYNKEENPRVPIIVTGNDFSTLYAPL

Creosote GRMGGTTQYTVNNQMVNATLMNIADNPTNVQLPGMYNKEENPRVPIIVTGNDFSTLYAPL

****.***************:**** ************::* **** *************

Spinach IRDGRMEKFYWAPTREDRIGVCTGIFKTDKVPAEHVVKLVDAFPGQSIDFFGALRARVYD

Picea IRDGRMEKFYWAPTRDDRIGVCQGIFRADNVHPDDVVRLVDTFPGQSIDFFGALRARVYD

Tobacco IRDGRMEKFYWAPTREDRIGVCTGIFRTDNVPAEDVVKIVDNFPGQSIDFFGALRARVYD

Hairgrass IPDGRMEKFYWAPTREDRIGVCKGIFQTDNVSDESVVKIVDTFPGQSIDFFGALRARVYD

Cotton IRDGRMEKFYWAPTRDDRIGVCKGIFRTDGVRDEDIVKLVDTFPGQSIDFFGALRARVYD

Creosote IRDGRMEKFYWAPTREDRIGVCKGIFRTDNVADDDIVKLVDTFPGQSIDFFGALRARVYH

* *************:****** ***::* * : :*::** *****************.

Spinach DEVRKWVNSVGVDNVGKKLVNSKDGPPVFEQPEMTLQKLMEYGNMLVQEQENVKRVQLAD

Picea DEVRKWVAGTGIQNIGKKLINSKEGPPTFEKPAMTIEKLLEYGNMLVGEQDNVKRVQLAD

Tobacco DEVRKWVSGTGIEKIGDKLLNSFDGPPTFEQPKMTIEKLLEYGNMLVQEQENVKRVQLAD

Hairgrass DEVRKWVSSTGIENIGKRLVNSRDGPVTFEQPKMTVEKLLEYGHMLVQEQDNVKRVQLAD

Cotton DEVRKWISEVGVASVGKKLVNSREGPPTFEQPKMTIEKLLEYGNMLVAEQENVKRVQLAD

Creosote DEVRKWVSEVGVDTIGKKLVNSKEGPPSFEQPKMTIDKLLGYGGMLVQEQENVKRVQLAD

******: .*: .:*.:*:** :** **:* **::**: ** *** **:*********

Spinach QYMSSAALGDANKDAIDRGTFFGKAAQ--QVSLPVAQGCTDPEAKNYDPTARSDDGSCTY

Picea KYMSEAALGDANEDSIKRGTFYGQAAQ--HVNLPVPEGCTDPRAANYDPTARSDNGSCEY

Tobacco KYLKEAALGDANADAINNGSFFAS------------------------------------

Hairgrass TYMSQAALGDANKDAMKTGSFYGKGAQ--QGTLPVPEGCTDRDAKNFDPTARSDDGSCLY

Cotton KYLSEAALGEANEDSINRGTFYGKAAQ--QVGVPVPEGCTDPNADNFDPTARSDDGTCTY

Creosote KYMSEAALGDANNDAIKRGTFYGGQAAQQVGNVPVPEGCTDPQATNYDPTARSDDGSCVY

*:..****:** *::. *:*:.

Spinach NL---

Picea EFKEQ

Tobacco -----

Hairgrass TF---

Cotton KF---

Creosote KF---

Fig S1: Alignment of sequences from spinach (*Spinacia oleracea* P10871.2), tobacco (*Nicotiana tobacum* NP_001312984.1), Sitka spruce (*Picea sitchensis*, ABK24548.1), Antarctic hairgrass (*Deschampsia antarctica*, AAP83927.1), cotton (*Gossypium hirsutum*, AAG61121.1), and creosote (*Larra tridentata*, Q7X9A0.1). The alpha isoform contains an extension at the C-terminal region, as indicated in grey.


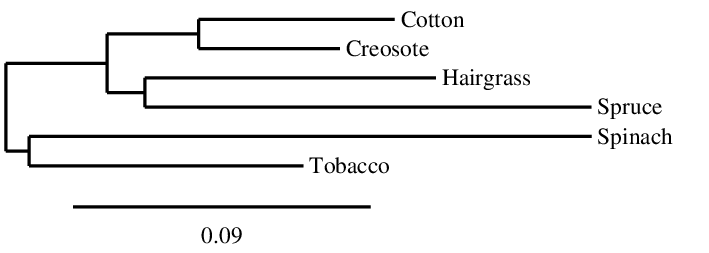


Fig S2: Phylogenetic tree generated from the alpha subunits from spinach (*Spinacia oleracea* P10871.2), tobacco (*Nicotiana tobacum* NP_001312984.1), Sitka spruce (*Picea sitchensis*, ABK24548.1), Antarctic hairgrass (*Deschampsia antarctica*, AAP83927.1), cotton (*Gossypium hirsutum*, AAG61121.1), and creosote (*Larra tridentata*, Q7X9A0.1).


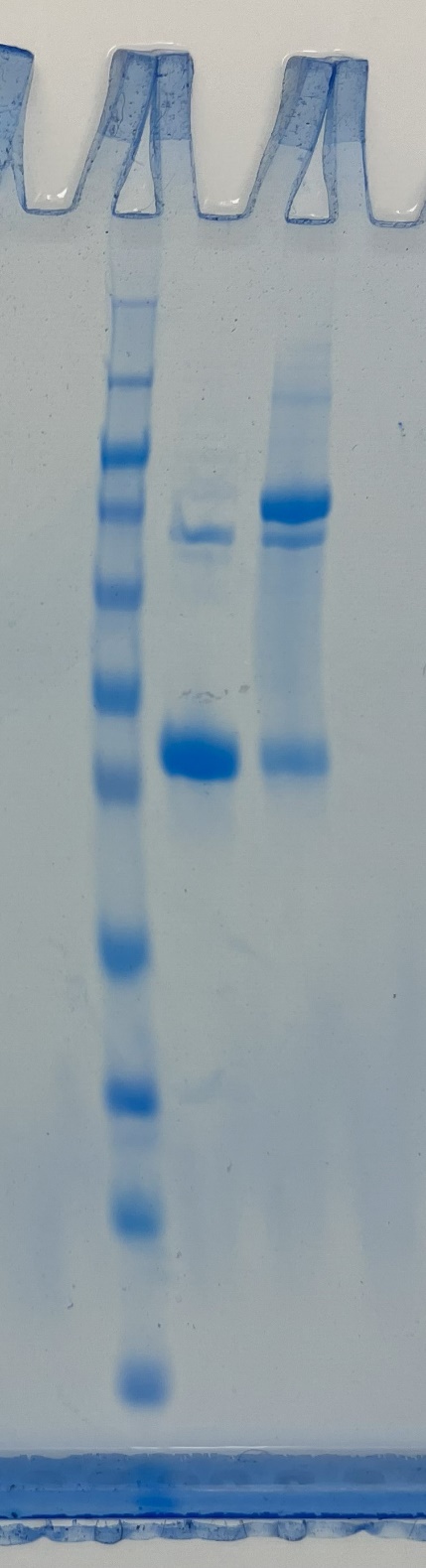


Fig S3: SDS-PAGE of the β-isoform of Sitka spruce Rca. The sample on the left was treated with 5 mM DTT before being run on the gel, while the sample on the right was not treated with DTT.
